# A scoping review of school-based indigenous substance use prevention in preteens (7–13 years)

**DOI:** 10.1186/s13011-020-00314-1

**Published:** 2020-10-01

**Authors:** Geoffrey Maina, Maeve Mclean, Solomon Mcharo, Megan Kennedy, Joseph Djiometio, Alexandra King

**Affiliations:** 1 College of Nursing, University of Saskatchewan, 210-1301 Central Avenue, Prince Albert, S5V 4W1 S.K Canada; 2 Master of Public Health, University of Saskatchewan, Saskatoon, Canada; 3 College of Nursing, University of Saskatchewan, Saskatoon Campus, Saskatoon, Canada; 4 Librarian- Library, and Museums - Public Services 2, University of Alberta, Edmonton, Canada; 5 Daphne Cockwell School of Nursing, Faculty of Community Services, Ryerson University, Toronto, Canada; 6 Cameco Chair in Indigenous Health, University of Saskatchewan, Saskatoon, Canada

**Keywords:** Substance use prevention, School-based, Indigenous children, Early intervention, Cultural appropriateness

## Abstract

**Background:**

Early-onset substance use is a risk factor for continued use, dependency, and poor long-term health outcomes. Indigenous youth are more likely to engage in early-onset substance use than their non-Indigenous counterparts. In Canada, culturally appropriate prevention programs are needed for Indigenous youth in elementary schools. Therefore, this scoping review aims to explore the published, international literature examining school-based substance use prevention programs for Indigenous children aged 7–13.

**Main text:**

*Methods:* This scoping review followed a six-step approach: 1) identifying the research questions, 2) identifying relevant studies, 3) selecting the studies, 4) charting the data, 5) collating, summarizing, and reporting the results, and 6) consulting with experts. The review was reported using guidelines from Preferred Reporting Items for Systematic reviews and Meta-Analyses extensions for Scoping Reviews (PRISMA-ScR). *Results:* Eleven articles (3 Canadian; 7 American and; 1 Australian) were included in the review. The prevention programs they studied were based on existing research or were adapted from existing interventions. The programs were tailored to each communities’ culture by including Indigenous stakeholders in developing or adapting prevention programs to be culturally safe and responsive. The articles evaluated the programs’ Effectiveness in changing student knowledge, attitudes, and behaviors using pre- and post-intervention surveys, randomized control trials, longitudinally designed analysis, and mixed methods. Mixed quantitative findings and qualitative findings highlighted the programs’ value in building community capacity and fostering cultural revitalization.

**Conclusion:**

This review highlights best practices for developing school-based substance use prevention programs for Indigenous youth. Findings suggest that prevention programs should be culturally responsive and provide students with the knowledge and skills to prevent and manage substance use in real-life situations. Making Indigenous beliefs, values, languages, images, and worldviews central to the prevention curriculum enhanced the Effectiveness, appropriateness, and sustainability of prevention programs. Indigenous communities are best positioned to facilitate cultural tailoring without compromising the fidelity of evidence-based prevention programs.

## Background

The current opioid overdose crisis, the harms caused by alcohol, and the new reality of cannabis legalization in Canada justify the effort to reassess substance use and their implications for public health [1]. Although alcohol is the most common substance used by Canadian youth in grades 7 to 12 [2, 3], its use by elementary school children is under-researched [4]. Between 20 and 50% of children aged 8–10 years have reported consuming an alcoholic drink recently [1, 4]. Cannabis is the second most used substance by elementary school children [2, 5]. Canadian youth have one of the highest rates of cannabis use worldwide, ranking first among 43 countries and regions across Europe and North America, with 33% of youth have tried cannabis at least once by the age of 15 years [6].

Inhalant abuse, which entails inhaling volatile substance to alter mental state is of major concern among children and youth [between 10 and 16 years] in Canada [7]. Inhalants are abused through sniffing, huffing or bagging and are often used for experiential, recreational or habitual reasons [8]. It is estimated that between 1 and 3% of children and youth seeking addiction treatment are due to inhalant abuse [9].

Substance use at a young age is a primary risk factor for continued use and dependence and has long-term health impacts [4, 10–13]. Early alcohol use has been linked to binge drinking, heavy drinking, and alcohol-related problems later in life [10, 14, 15]. Adolescent cannabis use interferes with healthy brain development, causing neurological impairment and mental health problems [10, 13, 16]. Substance use in youth also causes bodily harm arising from impaired driving, violence, sexual assault, unprotected sex, unwanted pregnancies, and educational failure [6, 13, 17, 18]. The multiple harmful effects of early initiation of substance use underscore the importance of prevention, early detection, and treatment intervention targeting youth [16]. This includes delaying substance use debut and reducing the negative social and behavioral consequences associated with underage substance use [12].

Like other Indigenous people around the world, First Nations, Métis, and Inuit face significant challenges with trauma – cumulative throughout an individual’s life, as well as collective (encompassing that experienced by one’s family, community, nation, as well as the surrounding natural world) and intergenerational – that leads to substance use and Addiction. Therefore, trauma is a determinant of health and health behaviors [19]. Also, alcohol and even substance use was not historically part of many Indigenous cultures or their protective societal fabric. It was introduced as part of colonization and used quite deliberately to increase colonizer’s profits and control [20]. See, for example, substance use, and addiction [21]. Indigenous people in Canada report higher rates of substance-related hospitalizations and overdoses than the general population [22]. The high incidence of substance use among this population has been attributed to the legacies of colonization, which alienated Indigenous population, suppressed their cultural beliefs and practices, created isolated reserves, and geographically segregated Indigenous population urban areas [23].

Also, the residential school program, which was meant to assimilate Indigenous Canadians into the mainstream culture, separated children from their families, where they experienced psychological and physical abuse. Both those who attended residential schools [24, 25] and subsequent generations [25, 26] have high rates of psychiatric problems and substance misuse. A study in British Columbia [27] found that 78.8% of residential school attendees reported have a substance use disorder, which means that residential school attendance remains a significant risk factor for substance misuse even when controlling for other factors, such as gender, age, and child abuse [27, 28].

Intergenerational trauma, also referred to as cumulative or historical trauma, refers to the cumulative emotional and psychological harm experienced throughout a person’s lifespan and across subsequent generations. Intergenerational trauma is a direct consequence of residential schools which signifies the internationalization of colonization [29] parents and families of children taken to residential schools. Research suggests that residential school survivors may express their grief as lateral violence towards family and community members, which often resembles experiences of abuse at residential school [24, 28, 30].

Due to the legacies of colonization, Indigenous children are more likely to experience adverse childhood experiences, such as violence and abuse, which are significant risks for substance use [24, 31, 32]. While Indigenous people in Canada are largely overrepresented in the addiction treatment system, there is a lack of recognition that Indigenous clients require culturally appropriate substance use treatment services [33]. Indigenous cultural beliefs and healing traditions are often excluded from substance use treatment services or social services [34, 35]. Consequently, Indigenous people tend to underutilize mental health and addiction treatment, because Addiction is seen and spiritual wounds. Hence, they are more likely to drop out of addiction treatment, especially if they are not culturally safe [36, 37].

Indigenous youth in Canada are more likely to engage in early-onset substance than non-Indigenous Canadian youth [38]. A 2008 British Columbia Adolescent Health Survey found that 42% of Indigenous youth had experimented with alcohol at age 12 or younger and that 28% of Indigenous youth living on-reserve experimented with alcohol at the age of 10 or younger [39]. Moreover, Whitebeck and colleagues [40] found that more than one-quarter of Indigenous youth aged 10–12 years old report substance use, which is twice the rate of non-Indigenous children. A study on sociodemographic characteristics and clinical outcomes for clients on opiate replacement therapy at a western Canadian clinic showed that 96% of the clients—the majority of whom are Indigenous—started using alcohol and marijuana at 6 and 7 years respectively [41]. This study inspired this scoping review aimed to identify substance use and addiction prevention for children between the ages of 7–13 years. Since this aga group is in elementary school, we believe that substance use schools are ideal settings for substance use prevention interventions. Substance use Focusing on substance use and addiction prevention for this group is necessitated by the need to understand ways to protect this demographic from early exposure to substance use which is a significant risk factor for the development of Addiction.

## Methods

This study utilises a scoping review approach to knowledge synthesis to determine the scope and coverage of literature on Indigenous school-based substance use prevention interventions for children aged 7–13 years. Unlike systematic reviews, scoping reviews are not aimed at confirming or refuting practice based on available evidence, or to establish the quality of evidence or to address uncertainty in practice [42]. Rather scoping review helps the researcher to obtain information on the volume of literature, studies available, and the overview of their focus [43]. The scoping review methodology is very useful for investigating the extent of the research in each topic area. This type of synthesis review methodology was selected because of its rigorous and methodical approach that allows for openly framed research questions. Further, it was important that this review not be limited to only one evidence type (e.g., only randomized controlled trials), as our preliminary search determined that the literature included a variety of qualitative evidence sources.

To ensure accurate and thorough reporting, the scoping review was conducted using the six-stage approach described by Arksey and O’Malley [43] and refined by Levac, Colquhoun, and O’Brien [44]: (1) identifying the research question(s), (2) identifying relevant studies, (3) selecting the studies, (4) charting the data, (5) collating, summarizing, and reporting the results, and (6) consulting with experts. The scoping review was reported using guidelines from the Preferred Reporting Items for Systematic reviews and Meta-Analyses extension for Scoping Reviews (PRISMA-ScR) a tool that is used to guide the scoping review process [41]. Also, to ensure methodological rigor, a scoping review protocol for the review was peer-reviewed and published in the journal, B.M.J. Open [45].

### Stage 1: Identifying the research questions

The scoping review aimed to explore current school-based interventions for the prevention of substance use in Indigenous communities. Consultations with a health science librarian (MK) helped to identify essential vocabulary that represented the broader focus of Indigenous elementary-school-based substance use prevention. We identified one broad research question: What is known about Indigenous elementary-school-based interventions for preventing substance use? We focused on this population because Maina Crizzle, Maposa, and Fournier’s research indicated that clients with addictions were often exposed to substances at a very young age [41]. M.K. ran a trial search to determine the feasibility of this research question. Following Arksey and O’Malley [43], our research question was refined as we became more familiar with the literature. Aiming to carefully examine and map the evidence on school-based interventions for preventing substance use in Indigenous children ages 7–13, we developed the following research questions:

1. What is known about Indigenous-focused, elementary-school-based interventions for preventing substance use?
2. What are the characteristics and outcomes of elementary-school-based interventions for preventing substance use?

### Stage 2: identifying relevant studies

Because M.K. was familiar with evidence synthesis and reporting, she guided the team throughout the search process to identify appropriate keywords and controlled vocabulary terms where available. Controlled vocabulary terms were used to enhance the search’s comprehensiveness and specificity. The following databases were searched using a combination of keywords and controlled vocabulary terms: MEDLINE in-process and other nonindexed citations (Ovid), 1946–present; PubMed, 1966–present; E.M.B.A.S.E. Classic (Ovid), 1947–present; Cumulative Index of Nursing and Allied Health Literature (C.I.N.A.H.L.) (E.B.S.C.O.), 1937–present; Educational Resources Information Center (ERIC) (Ovid), 1965–present; Scopus (Elsevier), 1970–present; and Cochrane Library (Wiley) and PsycINFO, 1806–present. See Table 1 for our full MEDLINE search strategy. In addition to searching electronic databases, we used the Canadian Agency for Drug and Technology in Health’s (C.A.D.T.H.’s) [61] Grey Matters tool to search for relevant grey literature. The C.A.D.T.H. tool searched resources such as Health Technology Assessment agencies (Canada, Australia, and the U.S.A.), free and subscription-based databases, and internet search engines. Keywords identified throughout our search process were used in the search process. We also checked the reference lists of the included studies to identify any resources that had not been found in our other searches.

**Table 1:** A summary of the articles that were included in the scoping review

S.No	Author	Country	Objectives	Target grades	Intervention	Cultural tailoring
1.	Asdigian et al. (2018) [46]	United States	To evaluate the Effectiveness of the Circle of Life (CoL) program in reducing marijuana use among Alaskan Native (AN) youth.	Grade 7–8	The Circle of Life (CoL) program is a 30-h health education and youth development curriculum that integrates theories of behaviour change and cultural knowledge, values, stories, illustrations, historical practices and teachings.	The Circle of life was developed by Alaskan Native educators and reviewed by parents, education specialists, and health experts from a wide range of AN communities and organizations.
2.	Baydala et al. (2009; 2014) [47, 48]	Canada	a) To describe the collaboration between the Alexis Nakota Sioux Nation and the University of Alberta to adapt, deliver and evaluate the first year of Life Skills Training program. b) To outline the process of culturally adapting, delivering and evaluating a substance abuse prevention program for school-aged children in the Alexis Nakota Sioux Nation.	Grade 3–9	The Life Skills Training (L.S.T.) program includes three levels, with each level consisting of between 8 and 14 one-hour lessons delivered to students. It consists of three modules, that consist of between 8 and 14 one-hour sessions delivered over the course of 3 years	In the first phase, school personnel, community stakeholders evaluated the program to make sure it reflected the traditional ways of knowing. In the second phase of the project, cultural adaptation of the L.S.T. program was achieved by a) establishment of an Adaptation Committee; b) incorporating traditional knowledge offered by the elders including cultural terms and cultural practices.
3.	Baydala et al. (2016) [49]	Canada	a) To culturally adapt Life Skills Training program to reflect the language, culture and visual images of Maskwasis community b) To deliver the adapted program in Maskwasis schools c) Evaluate the impact of the adapted program	Grade 3–8	The Life skills training is an 8-module program offered at three levels at elementary and junior high. It consists of 8 modules delivered as an initial lesson in the first year and booster sessions in the subsequent years. The intervention entails the incorporation of Indigenous and western foundations of substance use prevention.	Community elders were involved in adaptation of the original LST program which entailed incorporation of Cree language and syllabics, elders teaching, and personal life stories. Community members created visual images for manuals that reflect the Maskwasis culture and community.
4.	Hodder (2017) [50]	Australia	To examine the Effectiveness of a school-based resilience intervention in reducing the use of tobacco, alcohol and illicit substance use, and increasing individual and environmental protective factors among secondary high school students.	Grades 7–10	Each intervention school chose 16 broad strategies seeking to build protective factors across three domains: 1) curriculum, teaching and learning, 2) ethos and environment, 3) Partnerships and services.	Not applicable
5.	Johnson et al. (2009) [51]	United States	To examine the efficacy of the Think Smart school-based drug prevention curriculum among elementary school students	Grades 5–6	Think Smart is an adaptation of the Personal Intervention Curriculum for Native American adolescents that focusses on teaching a) drug refusal skills, b) anti-drug norms, c) personal self-management skills, and, d) general social skills to resist drug offers.	Researchers, and Alaskan natives and other Alaskan consultants were involved in the curriculum adaptation at three levels- surface, deep, and evidential levels. Surface adaptations included Alaksa-specific visuals and examples, deep adaptations involved integrating the values of Alaskan curricula, and evidential adaptations provided more Alaskan-specific statistics. Findings from a feasibility study also informed cultural adaptations.
6.	Kulis et al. (2017) [52]	United States	To describe a small efficacy trial of the Living in 2 Worlds prevention curriculum	Grades 7–8	Living in 2 Worlds (L2W) is a culturally adapted version of keepin’ it REAL (KiR) that teaches youth skills to resist drug offers, conduct risk assessments, improve decision making and develop other life skills.	The L2W was adapted using a community-based participatory research approach that employed expert knowledge of urban American Indian youth, parents, professional, and prevention curriculum specialists.
7.	Lowe et al. (2012) [53]	United States	To evaluate an innovative school-based cultural intervention targeting substance use among a Native American adolescent population.	Not specified	The Cherokee Talking Circle (C.T.C.) is a 10-session manual-based intervention where students engage in a 45-min talking circle led by a counselor and cultural expert, once a week over a 10-week period.	The research team established partnerships, steering committee which reviewed the intervention manual and selected the most culturally appropriate measures for substance a abuse. Adolescents provided feedback and recommendations that were addressed by the steering committee.
8.	Helm & Okamoto. (2013) [54]; Okamoto et al. (2012) [55]; Okamoto et al. (2016) [56]; Okamoto et al. (2019) [57]	United States	a) To outline collaboration among Hawai’ian Island communities and a university-based research term to develop, implement and evaluate the Ho’ouna Pono substance use prevention curriculum. b) To adapt and validate narrative scripts to be used for the video components of a culturally grounded drug prevention program for rural native Hawaiian youth. c) To examine the Effectiveness of the Ho’ouna Pono curriculum in reducing substance use, increasing the use of drug resistant strategies and improving psychosocial risk factors among middle, intermediate or multi-level school students d) To outline the drug use outcomes in an efficacy trial of a culturally grounded, school-based substance use prevention curriculum in rural Hawai’i	Grades 6–8	Ho’ouna Pono curriculum is a video enhanced curriculum is delivered once per week for 7 weeks. For each lesson, a video is shown depicting a drug offer, and three possible drug refusal options. Critical thinking skills and practical activities are included in the intervention.	Scripts were based on a multiyear study examining the most frequently experienced and challenging problem drug situations reported by rural Hawaiian youth. Youth identified the types of situations where drugs were offered, and the types of drug refusal strategies used. Scripts were based on the seven most frequently experienced and challenging problem drug situations reported by rural Hawaiian youth.
9.	Stanley et al. (2018) [58]	United States	To present research findings being used to adapt an existing substance use prevention media campign for American Indian youth.	Grade 7 and 11	The Be Your Own Influence (B.U.Y.O.I) is a media-based substance use prevention campaign that target middle school youth to reframe substance use that is inconsistent with personal autonomy and aspirations. It emphasizes the prosocial influence of older peers at a time when students begin to make decision and substance use.	Focus groups were carried out with 7th graders, and photovoice was carried out with 11th grades to guide cultural adaptations.
10.	Usera et al. (2017) [59]	United States	To examine the Effectiveness of the Lakota Circles of Health (L.C. H) in improving healthy decision making on substance use, conflict resolution, communication, self-identity and cultural competence among elementary school students in four American Indian reserves.	Grades 4–5	The Lakota Circles of Hope (L.C. H) is a substance use prevention program delivered through grades 2 to 5. It consists of 10 lessons per school year that is based on making healthy decisions within the context of Lakota traditions and values.	The L.C.H. was adopted by a team of Lakota educators and was based on four Lakota values: generosity, courage, wisdom and respect. Educators identified instructional patterns, strategies and performance outcomes for the delivery of lessons and activities. Each lesson was adapted to incorporate Lakota ways of knowing and being.
11.	Wexler et al. (2017) [60]	United States	To describe the process and outcome evaluation of the Youth Leadership Program in rural Alaskan schools.	Grade 3–12	The Youth Leadership program was adapted from the Health Education Foundation’s Natural Helper curriculum. It uses helpers and peer leaders to increase protective factors and to reduce risk factors associated with drug use, alcohol use, violence and bullying.	The curriculum was previously adapted to reflect the realities of the school district and the cultural norms of Alaskan native youth. It incorporates Inupiaq cultural values such as respect for others, cooperation, hard work, responsibility to the tribe, and sharing.

The results from each database search were documented and saved, and references were imported into EndNote, a bibliographic management software. Following the removal of duplicate references, references were imported into Rayyan, a review software [62], for the title and abstract screening. Google Forms was used to collect data during full-text analysis. To facilitate the retrieval of relevant articles, the search was limited by language (English) and publication date (2009–2019). See below for inclusion/exclusion criteria.

### Inclusion criteria

The following 4 inclusion criteria were identified and used to guide the searches and review the articles: First, only English-language articles published between 2009 and 2019 were included. As a relatively new methodology, there is no consensus on the publication age range of the articles to be included in a scoping review [63]. Publication age range of articles included in the scoping reviews have ranged from less than 5 years to no limit on dates [64, 65]. Arksey and O’Malley [43], whose methodological framework is used in this scoping review do not recommend publication date range. Rather, they propose the reason for counting the scoping review be the guiding principles for researchers conducting a scoping review. Research team consensus was that a 10-year publication age range for including articles for this review was adequate to address the research question. Second, the studies needed to identify Indigenous children ages 7–13 living in Canada, Australia, New Zealand or the United States, (C.A.N.Z.U.S.) as one of the target populations. These countries were considered because although they are first world countries, their Indigenous population have similar histories of colonization and whose impact is comparable. Third, studies that comment on mixed-age populations of Indigenous children and adolescents were included for text analysis to further explore their suitability for inclusion. Fourth, the studies needed to discuss or present a school-based substance use prevention intervention for this target population or the intervention’s result.

### Exclusion criteria

The following exclusion criteria were identified: 1) articles discussing interventions for addictions such as smoking, gambling, internet/social media/technology, [cigarette smoking was not included in this review because it was not identified in the project that inspired this scoping review as a risk that children aged 7–13 years engaged] 2) articles discussing adult populations (ages 18+), and 3) review articles and commentaries.

### Stage 3: study selection

Rayyan [62] was used for the title and abstract screening process. We identified inclusion and exclusion keywords in correlation with identified inclusion and exclusion criteria. For example, exclusion keywords for smoking, gambling, etc. were identified in exclusion criterion 1. Two reviewers independently reviewed all articles and a third reviewer was used to reach consensus when necessary. This process was repeated during the full-text analysis when we used EndNote to record data and review the full-text articles. Google Forms was used to collect data (see below, “Charting the data”).

### Stage 4: charting the data

We extracted the following review data into a Google survey: author(s), age groups of the target population, the country of residence; summary of intervention characteristics that included cultural tailoring.

### Stage 5: collating, summarizing, and reporting the results

The purpose of a scoping review is to map and aggregate findings to present an overview of the topic. Accordingly, we collected data to map the results (main sources, locations, and quantity), provide a descriptive summary and qualitative analysis, identify conceptual definitions, provide a glossary of terms to clarify definitions found in the literature, and report our results using the PRISMA-ScR guidelines to enhance transparency and reproducibility. Because this is a scoping review, we did not appraise the quality of the studies or offer statistical analysis. Thematic analysis was applied to identify common threads that emerged from the data.

### Stage 6: consulting

We will present our preliminary findings of this scoping review to an advisory group comprised of First Nation Elder from Treaty 6 Territory, a Knowledge Carrier, elementary school leadership, and community members that G.M. has a collaborative relationship with, and one whose children are impacted by substance use. Community partners working with G.M. will help to identify the appropriate advisory team members to join the committee. Presenting the findings to this committee will help validate the results and provide a basis for reflection and feedback on the relevance of similar interventions in the community. Their feedback will inform the discussion, recommendations, and implications for the practice section of the review. Approval from the Research Ethics Board was not required to complete this project**.**

## Results

**Fig. 1:**
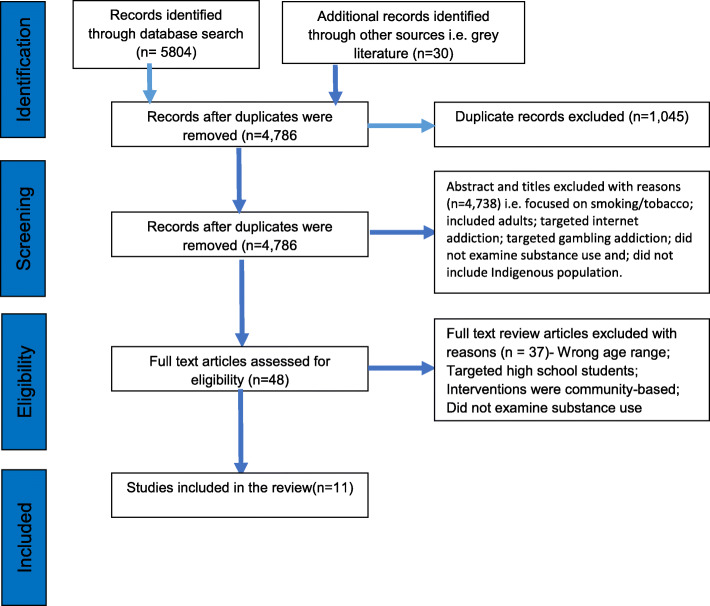
A flow chart- prisma diagram that illustrates the steps and outcomes of the scoping review

Eleven projects were included in the review, of which seven of them were based in the United States, and three were based in Canada and one in Australia [Fig. 1, P.R.I.S.M.A. diagram]. The interventions focused on substances—drugs, alcohol, tobacco, and legal products that could be harmful—and skill development—resilience, making healthy choices, promoting protective factors, drug-refusal skills, and reducing risk. The review is organized in four interlinked thematic areas are the project overview; how the project was made culturally safe; the project intervention strategy; and the project evaluation mechanism. [Table 1, article summaries].

### The project overview

The projects in this review developed from bottom up or were adapted from existing programs. Baydala et al. [47] aimed to describe the adaptation process for alcohol and substance use prevention programs for elementary school children. Their project was adapted from the life skills training program, existing school alcohol, and substance use prevention program. The program was chosen because of its effectiveness in randomized control trials. Baydala, 2014 [48] builds on Baydala et al. [47] and describes the process of culturally adapting, delivering and evaluating a substance use prevention program for school-aged children in the Alexis Nakota Sioux nation. Baydala et al. [49] on the invitation of the Maskawasis four nations partnered to culturally adapt, implement and evaluate the life skills training program. The researchers and the community partners endeavoured to preserve the fidelity of the original curriculum and document the impact of the adapted curriculum. Hodder et al. [50] selected a pragmatic school-based universal resilience intervention to reduce the prevalence of tobacco, alcohol, and illicit substance use, and to increase individual and environmental factors that prevent students’ drug use. Each school chose and implemented programs that had 16 broad strategies that supported the desired outcome without making any modification.

Wexler et al. [60] implemented the Youth Leaders Program, a modification of the Comprehensive Health Education Foundation’s Natural Helper curriculum. The Youth Leaders Program aimed to reduce youth suicide by attending to associated risk factors, such as substance use and bullying, and by increasing protective factors, such as school attendance and engagement, and academic success. Johnson, Shamblen, Ogilvie, Collins, and Saylor [51] adapted their Think Smart program from substance use prevention curricula for the Indigenous adolescents, which were based on the cognitive-behavioral model. This model emphasizes personal self-management skills and the social skills to refuse drugs and to reduce motivation to use drugs. Kulis, Ayers, and Harthun’s [52] project, Living in 2 Worlds (L2W), was adopted from the Keeping it Real program (KiR), a universal school-based substance use prevention program that teaches youth skills to resist substance use through the refuse, explain, avoid, and leave (R.E.A.L.) strategy. L2W was redesigned for urban American Indian middle-school students and focused on strengthening resilience and decision-making skills using a culturally grounded prevention message.

Usera’s [59] Lakota Circle of Hope (L.C. H) curriculum was adapted from the existing program for children and entails age-appropriate topics, medically accurate information, and culturally influenced prevention instruction. The curriculum was designed to reduce early risky behaviors by using an appreciation of Lakota values and traditions as a framework for making decisions and choices that contribute to a healthy and safe environment. It aimed to equip students with the skills to make healthy decisions in the context of Lakota traditions and values within a school environment.

Helm and Okamoto [54] developed a video-enhanced, classroom-based curriculum, called Ho′ouna Pono based on the findings their research into drug offers and refusal for students. They then created interventions that depicted scenarios of drug offers and drug-refusal options. Diverse publications emanating from this work have been made focussing on; a) description of the process of creating the videos [Okamoto, Helm, McClain, and Dinson’s [55]; b) efficacy trial of the intervention [Okamoto, Kulis, Helm, Chin, Hata, Hata, et al. [57] and; c) evaluation of the curriculum. [Okamoto, Kulis, Helm, Lauricella, Valdez, [56].

Asdigian, Whitesell, Keane, Mousseau, and Kaufman [46] evaluated the Circle of Life (CoL) program’s Effectiveness in reducing marijuana use among elementary school children in a rural, Northern Plains reservation. They chose this program because of its previous Effectiveness in delaying sexual initiation.

Stanley, Kelly, Swaim, and Jackman [58] described the adaptation process for the Be Under Your Own Influence (B.U.Y.O.I) media campaign for American Indian youth. It was a media-based substance use prevention campaign developed through extensive formative research and evaluation using reframing theory and theory of reasoned action. Randomized control trials showed that it reduced students’ marijuana and alcohol use.

Lowe, Liang, Riggs, and Henson [53] compare the Cherokee Talking Circle (C.T.C.), a culturally based intervention, to a standard substance use intervention revised from the Drug Abuse Resistance Education (D.A.R.E) program. The talking circle was adapted from the Cherokee self-reliance model that was developed from findings of studies that explored how the Keetoowah-Cherokee people conceptualized self-reliance. it was designed for Keetoowah-Cherokee students who were in the early stages of abusing substances and experiencing the negative consequences. The intervention’s goal was to reduce substance use, ideally to attain abstinence.

### How the projects were made culturally safe

The projects were culturally adapted to suit the communities’ needs either by modifying the original programs or by consulting the communities during program development. The project adaptation for Baydala et al. [47] began by translating the original program to the Isga language (the community’s ancestral language) and then back-translation to English. Indigenous ways of knowing and practices, such as ceremonies, prayers, storytelling, circle theories, lived experiences, and cultural activities, were incorporated into the curriculum. A community artist created culturally appropriate images to replace those of the original program. A program naming ceremony was also held before the program was launched. Baydala [49] adapted life skills training program, a generic proven highly effective program for substance use prevention for students of diverse geographical and socioeconomic background. The adaptation process by constituting an adaptation committee to review and recommend adaptations of the L.S.T. curriculum. The adaptation comprised of using Cree language and syllabics, infusing elders’ teachings and personal life stories and incorporation of visual images that reflected the Maskwacis culture and community.

To create the Ho′ouna Pono Drug Prevention curriculum [55–57] the narrative scripts’ settings, language, and behavior were changed to reflect the rural Hawaiian people’s worldview. The researchers consulted with community partners, including older adolescents, educators, parents, and professionals, to validate the drug prevention curriculum’s information so that the intervention reflected the community’s realities.

Johnson’s et al. [51] Think Smart project [46] used three types of cultural adaptations i.e. surface adaptation, (which entails use of idioms, language, and phraseologies used by the target group); deep adaptations (incorporating elements that express target populations’ culture, history, mores, physical environment and spirituality) and evidential adaptation(using empirical information of the target population).

The L2W [52] project was adapted using a theoretical model of cultural adaptation that entailed adding culturally appropriate language, images, scenarios, and formats for urban Alaskan Indian youth while maintaining the core components of the KiR program. It also integrated three types of information: 1) prior research on substance use risk and protective factors, 2) culturally specific ways urban Alaskan Indian youth encounter and resist substance offers, and 3) Indigenous cultural elements.

The Be Under Your Own Influence (B.U.Y.O.I) media campaign [58] underwent surface and deep adaptation. Surface adaptation entailed replacing the original campaign images with those that reflected American Indians and their environment. Deep adaptations created messaging focused on finding strength in one’s tribal history, culture, and identity. The intervention’s content focused on personal autonomy and aspiration. Three community advisory committees provided inputs on the cultural adaptations. Thereafter, two qualitative studies—a focus group discussion and a photo-voice project—were conducted with 7th-grade students and high school role models to obtain adolescent perspectives on issues of health and wellness and to assist in increasing the Effectiveness of the adapted intervention.

When developing the C.T.C. intervention, Lowe, Liang, Riggs, and Henson [53] formed a community partnership steering committee whose meetings were led by a Keetoowah-Cherokee elder and included six Keetoowah-Cherokee community representatives. During the intervention, the student participants engaged in a group led by a counselor and a cultural expert. The intervention manual was written in both English and Cherokee.

### Project interventional strategy

Two strategies were employed to develop and implement youth substance use prevention programs: integrating the intervention curricula with the regular school-based program or running it as a stand-alone program.

Baydala et al. [47, 49] designed their project as a 3-level curriculum, starting with level 1 for either grade 3 or grade 6 classes, which would run for 3 years. The first year has 8–14 sessions, and subsequent years had 8–10 booster sessions. The program was delivered once a week as part of a regular school curriculum for 2 h by a trained community program provider.

Ho′ouna Pono [54–57] was a classroom-based, video-enhanced curriculum for grades 6–8, and was comprised of 7 lessons delivered once a week. Each lesson began with a video depicting a drug offer and 3 possible drug-refusal options. The intervention was aligned with the state and national education standards for middle-school students emphasized critical thinking skills and taught key terms in drug prevention and Hawaiian culture. Drug-refusal skills were practiced through role-playing and small group co-learning.

The Think Smart project [49] curriculum consisted of 12 core sessions with 3 booster sessions 2–3 months later. The core sessions included sessions on stereotypes and drug factors, problem-solving models (stop, option, decide, act, and self-talk). The intervention emphasized refusal and self-assertiveness, 3 risks (refusal skills for, peer use of, and peer normative beliefs about harmful legal products) and 3 protective factors (knowledge about drugs and the consequences of drug use, assertiveness skills, and cultural identity). Core and booster sessions were taught weekly as a 1-h session to 5th and 6th-grade students by classroom teachers.

Hodder’s [50] 3-year universal intervention focused on grades 8–10 and involved 16 broad categories aimed at building protective factors across 3 domains of health promotion within a school framework. The schools were provided with details of existing resources and programs that they could choose to implement. These resources addressed one or more individual factor (self-efficacy, problem-solving, cooperation/communication, self-awareness, empathy, and goals/aspirations) or environmental factor (school support, meaningful participation in school, community support, meaningful participation in the community, home support, meaningful participation at home, caring peer relationships, and prosocial peers). The intervention was delivered by school staff as part of the routine school curriculum.

The L2W [52] project was a strength-based curriculum designed to encourage students to explore their heritage by integrating Indigenous cultural elements into their curriculum. Twelve structured sessions of approximately 45 min in length were taught weekly. A cultural heritage project was integrated into the curriculum, as was a KiR lesson with scenarios emphasizing the diverse cultural ways youth can use to refuse substance offers.

The L.C.H. curriculum [59] consisted of 10 45-min lessons per year for grades 2–5 on making healthy decisions informed by Lakota traditions and values within the school environment. It was based on Lakota values of generosity, fortitude, wisdom, and respect. Each lesson conveyed Indigenous culture through stories, crafts, activities, and content, which was then applied to students’ daily lives. Families and communities were embedded as major components of the curriculum.

Each school participating in the Youth Leader Program [60] selected 4 to 18 youth leaders by a vote. The students chosen attended weekly meetings to review the previous weeks’ events and plan for future work. Advisors—teachers or elders—oversaw the group’s work.

The CoL project [46] used a 30-h health education and youth development curriculum. It was developed by Indigenous educators and reviewed by parents, education specialists, and health experts. Its integrated theories of behavioral change into the curriculum, which was based on Indigenous cultural knowledge, values, teachings, imagery, and practices. The project emphasized the responsibility to one’s family and community and the role of the community in preventing H.I.V. and other diseases. The medicine wheel was also integrated into the curriculum. The activities were intended to empower support empowered students to take personal responsibility, build communication, make a smart decision, resist peer pressure, and learn refusal skills. The course was taught by community members who were qualified to teach.

The Cherokee Talking Circle (C.T.C.) [53] was a 10-session, manual-based intervention where students aged 13–18 met for 45-min talking circles once a week for 10 weeks. The talking circle was not integrated within the curriculum but was implemented in a classroom setting. The talking circle provided an appropriate setting for culturally based discussions about substance use because it allowed participants to come together, accept each other, and share stories respectfully.

### Project evaluation mechanism

Both qualitative and quantitative approaches were used to measure the interventions’ effectiveness through changes in students’ knowledge, attitudes, and practices. Baydala et al. [47] evaluated the project’s effectiveness by measuring the students’ knowledge of the negative effects of drug and alcohol use, attitudes towards drug and alcohol use, refusal skills, and life skills. Pre- and post-intervention surveys measured the changes in knowledge, while focus group discussions were completed with the school personnel, community members, and elders involved with the program. Most students increased their knowledge of drugs and life skills and increased their knowledge about drugs. The community found the program beneficial as it emphasized culture and tradition. However, the community also found the program time consuming as it brought a heavy workload and stress, partially caused by the complexity of translating the curriculum from English to Isga and back.

Baydala et al. [48] evaluated the Effectiveness of cultural adaptation in curriculum programming and the perceptions of the benefits and suggested improvements. They found that the project helped embody the community’s values by incorporating their language, history, culture, and wisdom. Moreover, students increased their cultural knowledge, changed their behaviors, and improved their self-esteem. Participating in the program also improved school attendance and increased students’ knowledge of alcoholism’s negative impacts and strategies to avoid risks such as drinking. To sustain the gains made by the in-school program, the community wanted to create a similar program for the wider community.

Baydala [49] evaluated program effectiveness by conducting 25 focus group discussions with students, elders, parents, facilitators, and school personnel. The F.G.D.s focussed on the program’s impact, factors that contributed to the program’s success, and suggestions for improvement. The program’s impact was felt in school as it allowed the elders to teach on culture and community knowledge. Students who participate in the program improved their self-esteem, and attitudes towards schools and the community changed. Through this program, elders were reminded about the ancestral teachings as they were shared by other elders. Focus on cultural teaching, and elder involvement was reported to be paramount to program success, and the program was also found to be relevant to the community to be introduced to the school. Facilitator skills and teacher involvement in the program enhanced overall success. Questionnaires were administered to the learning of program content, and the results showed an improvement in all the L.S.T. scores for elementary and junior high students on anti-smoking knowledge, and life skills knowledge.

Okamoto, Kulis, Helm, Lauricella, and Valdez [56, 57] conducted a randomized control trial to determine Ho′ouna Pono’s Effectiveness and used different data collection tools—sociodemographic items, risk and protective factors, drug resistance strategies, and risk assessment. Substance use changes over 2 years was assessed [52]. Findings from both studies [56, 57] supported the intervention’s effectiveness as youth exposed to the curriculum maintained or sustained drug resistance strategies.

Johnson, Shamblen, Ogilvie, Collins, and Saylor [51] conducted a randomized and matched control nested repeat measures analysis of youth to measure the ThinkSmart program’s Effectiveness in preventing youth’s use of legal products (inhalants, prescription medicines, over the counter medication, and common household products) that can be harmful such as to get high. In this project no adaptation was done. Findings indicated that the ThinkSmart curriculum significantly reduced the use of harmful legal products at 6 months and after completing the curriculum but had no impact on tobacco, alcohol, or marijuana use. Hodder et al. [50] conducted a cluster randomized control trial to measure the Effectiveness of the universal resilience intervention using pre- and post-intervention surveys as data collection tools. The study found no difference between the intervention and control groups for substance use. Kulis, Ayers, and Harthun [52] conducted randomized control trials to measure the Effectiveness of the L2W program and the KiR program. They found that the L2W group had more positive changes regarding cigarettes, alcohol, marijuana, and substance use.

Wexler et al. [60] used a mixed-methods approach such as surveys and focus group discussion to evaluate the process and outcome measures of the Youth Leaders without making adaptations to the research tools. The program positively affected participants: they reported an increased sense of agency, responsibility, and confidence. They also felt more mature, more willing to help, less shy, and more willing to speak up, and were thus able to make the school community more positive for the other students. Participating in the project significantly increased their school attendance and G.P.A., improved self-esteem, and reduce alcohol and drug intake. However, they faced challenges, such as competing obligations and the pressure to demonstrate exemplary behaviors.

Usera [60] used mixed methods to test the L.C.H.’s Effectiveness in reducing risky behaviors without making adaptations to the program. He used pre- and post-intervention surveys to collect data. The intervention group showed an improved understanding of Lakota values and traditions and a lower rate of alcohol use, marijuana use, huffing, and sex. Participants also acquired the skills to prevent disagreements from escalating into physical fights and bullying. Participants increased their self-esteem and self-efficacy and improved communication with adults.

Asdigian, Whitesell, Keane, Mousseau, and Kaufman [46] used group randomized control trials to evaluate the Circle of Life (CoL) program’s Effectiveness in reducing marijuana use without making adaptations to the evaluation tools. They collected baseline and current marijuana use information, which showed that youth who received CoL intervention were less likely to initiate marijuana use between 12 and 14 years of age. However, there was no evidence that the intervention affected the frequency of use for existing marijuana users.

Stanley, Kelly, Swaim, and Jackman [58] used focus group discussions and a photo-voice project to inform the program adaptation. Students chose photos that reflected the positive aspects of life on their reservation and text that conveyed good choices to reach goals and build a positive future. For the photo-voice project, the photos taken were categorized according to what inspired them to be drug-free (people, achievement, expression, culture, and traditions) and what made them unique (passions, hobbies, activities, style, and belonging to the tribe and its land).

Lowe, Laing, Riggs, and Henson [53] conducted a 2-condition, quasi-experimental design to compare a culturally based Cherokee Talking Circle (C.T.C.) intervention and standard education intervention. They collected data pre-intervention, immediately post-intervention, and 90 days post-intervention. They found that the culturally based intervention was significantly more effective for reducing substance use and related problems than the non-culturally based intervention for Indigenous adolescents. Furthermore, the C.T.C. groups had significantly better results over time than the non-culturally based standard education groups.

## Discussion

From this scoping review, researchers and community members either adapted a pre-existing intervention or developed a new program in response to identified community needs. Cost, time, and expertise are essential considerations for adapting a program or building one from bottom up [66, 67]. Program adaptation may be a preferred approach to implement an effective intervention to make it culturally appropriate for a community, when targeting a new population, or in a new community setting [68]. In adaptation to a program, implementers are confronted by the tension between the need to maintaining fidelity to the original program and meeting the identified community needs [69]. Nevertheless, an adapted program can realize outcomes that are comparable to the original program with proper planning, technical support, and training to program implementers [66, 70].

Developing an intervention from the bottom up can best be understood using community development model’s concepts of a) decentralization, which places planning and evaluation functions at a local level; b) participatory planning and implementation of programs which involve affected community setting their priorities and goals and c) multisectoral involvement that entails the inclusion of representatives from diverse sectors [71]. Thus, the affected community is involved at every stage of the program development, which distributes decision-making powers, empowers the community, and creates a cohesive community [72].

Early exposure to substance use increases the risks of alcohol dependence, development of blood-borne infection, early pregnancy, alcohol use during pregnancy, and engagement in illegal activities [73–75]. Interventions focusing on substance use prevention for Indigenous elementary school children are in response to a high prevalence of problematic substance use among Indigenous populations [76]. These interventions are based on the premise that preventing or delaying substance use debut may prevent, reduce, or delay risky behaviors later in life. This review provides insights into best practices that can be adopted in the development of culturally responsive substance prevention for Indigenous elementary students.

Elementary school systems are ideal settings to introduce substance use prevention interventions [77]. A school system can influence children’s behaviors and practices through knowledge impartation and socialization [78]. In addition, an intervention integrated with the school curriculum can reach many children, which can have a far-reaching effect, especially if they are taught by people they can trust. School settings are common places where experimentation with substance use happens driven by peer pressure [79]. The efficacy of most substance use prevention programs that are implemented in school settings has been established. These programs primarily equip the students with knowledge and skills to refuse drug offers, resist drug prodrug influences, enhance social and personal competent skills and to challenge the misperception of the normativity of drug use [79]. Such interventions also improve school outcomes such as attendance and overall success rates [80].

Making substance use prevention programs culturally safe is foundational to enhancing their effectiveness and acceptability among Indigenous populations. As experts in their own cultures, Indigenous stakeholders must be involved in developing, appraising, and adopting programs targeting Indigenous children. In so doing, they become partners, which enhances the program’s buy-in. Culturally responsive programs are designed to respond to the targeted population’s needs by adapting evidence-based treatments and ensuring that they are delivered by culturally competent providers [81, 82]. Culturally adapted evidence-based interventions better mitigate risks for substance use and increase the programs’ reception, acceptance, and salience [83–85]. In this review, programs’ cultural adaptation for Indigenous elementary children was a substantial undertaking that endeavored to make them resonated with the students’ lived realities. These adaptations included modifying pre-existing projects to reflect Indigenous lives to incorporate cultural beliefs, values, language, images, ways of knowing, and other forms of cultural representation [47, 51, 53, 55, 57, 73], or including Indigenous elements from the inception of the project [54, 58].

Community participation is critical to developing a culturally responsive program as community members ensure that the program is culturally safe and responds to the community’s needs. Community participation in the program development process ensures that the community’s values, beliefs, behaviors, norms, and worldview enhance the project’s acceptability and ownership [55, 57, 85]. Community partners are best placed to champion cultural tailoring—the act of incorporating cultural elements to the programs to address health disparity—to promote equity and address risks that increase their vulnerabilities to health inequalities because of their unique and deep understanding of their needs [66, 70]. Community experts can also provide substantive input on the adapted program’s structure and content without compromising the fidelity to the original program [47, 51, 73].

Equipping youth with knowledge and skills on how to manage risks and foster protective factors in real-life situations is critical [52, 55, 85]. Risk avoidance or risk management strategies can be achieved through providing social support, creating safe social networks, promoting self-efficacy, problem-solving, communication, and self-awareness, and fostering a supportive environment both at home and in the community [50, 59, 60]. These changes are vital for creating or fostering individual agency and responsibility. In addition, attending to mental health and wellness is vital for enhancing a sense of self-esteem, self-pride, identity, and purpose. Also, understanding that children at risk of early substance use may also be exposed to diverse adverse childhood experiences can be key to early screening for childhood trauma.

Understanding the project’s impact on the targeted community and the significant community involvement/ownership.is key to demonstrating evidence of program effectiveness. Pre- and post-intervention surveys [48, 51, 54, 59, 73, 74, 83], randomized control trials [50, 83], longitudinal design [61], and mixed methods [47, 50, 51] were deployed to provide empirical evidence of the interventions’ impact. Studies mainly assessed risk reduction, knowledge, and skills impartation. Qualitative data collection methods focused on understanding the program implementation processes and their impact on the community. Although there were mixed results regarding the programs’ impacts on the students’ knowledge of drugs and their skills to assess and avoid risks, the qualitative findings offered insights into the projects’ value, lessons learned, and challenges that the communities faced in carrying out the program. The accrued benefit to the communities included creating opportunities to foster cultural revitalization [48, 50] and granting personal satisfaction in integrating culture in substance use prevention.

The findings of this review may be limited by the nature of exclusion criteria applied to the articles included through language and publication date.

## Conclusion

It is commendable that programs endeavor to make early interventions to prevent substance use for elementary school children. While not always explicitly expressed in the articles reviewed, there was a sense that researchers and the communities had longstanding relationships that culminated in, among other things, the development and implementation of the interventions. Also, the interventions that were developed using bottom-up approaches, i.e., from foundational assessment data, took longer to develop due to the different stages required to validate them. Also, since most of the interventions were championed by researchers with affiliations with a university, it can be assumed that most of these projects were funded with research dollars. Taking these factors into consideration, it is necessary to attend to the financial sustainability of the intervention beyond the life of the research project. Besides, it is necessary to focus on building community capacity to sustain the intervention once the research project is over. Moreover, to sustain the gains that school interventions made, it is important to also develop similar interventions for families and communities.

## Data Availability

Not applicable.

## Abbreviations

PRISMA-ScR: Preferred Reporting Items for Systematic reviews and Meta-Analyses extension for Scoping Reviews
B.U.Y.O.I: Be Under Your Own Influence
L2W: Living in 2 Worlds
L.C.H: Lakota Circle of Hope
CoL: Circle of Life
C.T.C: Cherokee Talking Circle
R.E.A.L: Refuse, Explain, Avoid, Leave
